# Final outcomes of radial nerve palsy associated with humeral shaft fracture and nonunion

**DOI:** 10.1186/s10195-019-0526-2

**Published:** 2019-03-28

**Authors:** Rebekah Belayneh, Ariana Lott, Jack Haglin, Sanjit Konda, Philipp Leucht, Kenneth Egol

**Affiliations:** 1 NYU Hospital for Joint Diseases, 301 East 17th Street, New York, NY 10003 USA; 2 Jamaica Hospital Medical Center, Jamaica, NY USA

**Keywords:** Radial nerve palsy, Humeral shaft, Humeral fracture, Humeral shaft nonunion, Acute humeral shaft fracture, Radial nerve identification

## Abstract

**Background:**

Little evidence regarding the extent of recovery of radial nerve lesions with associated humerus trauma exists. The aim of this study is to examine the incidence and resolution of types of radial nerve palsy (RNP) in operative and nonoperative humeral shaft fracture populations.

**Materials and Methods:**

Radial nerve lesions were identified as complete (RNPc), which included motor and sensory loss, and incomplete (RNPi), which included sensory-only lesions. Charts were reviewed for treatment type, radial nerve status, RNP resolution time, and follow-up time. Descriptive statistics were used to document incidence of RNP and time to resolution. Independent-samples *t*-test was used to determine significant differences between RNP resolution time in operative and nonoperative cohorts.

**Results:**

A total of 175 patients (77 operative, 98 nonoperative) with diaphyseal humeral shaft injury between 2007 and 2016 were identified and treated. Seventeen out of 77 (22.1%) patients treated operatively were diagnosed preoperatively with a radial nerve lesion. Two (2.6%) patients developed secondary RNPc postoperatively. Eight out of 98 (8.2%) patients presented with RNP postinjury for nonoperatively treated humeral shaft fracture. All patients who presented with either RNPc, RNPi, or iatrogenic RNP had complete resolution of their RNP. No statistically significant difference was found in recovery time when comparing the operative versus nonoperative RNPc, operative versus nonoperative RNPi, or RNPc versus RNPi patient groups.

**Conclusions:**

All 27 (100%) patients presenting with or developing radial nerve palsy in our study recovered. No patient required further surgery for radial nerve palsy. Radial nerve exploration in conjunction with open reduction and internal fixation (ORIF) appears to facilitate speedier resolution of RNP when directly compared with observation in nonoperative cases, although not statistically significantly so. These findings provide surgeons valuable information they can share with patients who sustain radial nerve injury with associated humerus shaft fracture or nonunion.

**Level of evidence:**

Level III treatment study.

## Introduction

Humeral shaft fractures account for 1–3% of all skeletal fractures and demonstrate a bimodal age distribution, with average age of incidence greater than 50 years [1–3]. The majority of humeral shaft fractures present with a simple fracture pattern and are associated with low-energy injury mechanism. Ekholm et al. [1] conducted a review of 401 consecutive humeral shaft fractures, noting that that 61% were Arbeitsgemeinschaft für Osteosynthesefragen (AO)/Orthopaedic Trauma Association (OTA) type A, or simple fractures, and 68% were the result of a low-energy fall. Approximately 95% of humeral shaft fractures heal with nonoperative management [4–10]. Of fractures that require operative treatment, approximately 5–10% will result in nonunion [11–17].

Previous studies have demonstrated that radial nerve palsy occurs in between 2 and 17% of cases and is the most frequent nerve lesion following humeral shaft fracture [18]. Ekholm et al. [1] revealed the overall incidence of radial nerve palsy in closed humeral shaft fractures to be 8.5%. In a study featuring a prospectively collected database of over 5700 polytrauma patients, Noble et al. [19] not only concluded that the radial nerve was the most commonly injured peripheral nerve but that it had an overall incidence of 9.5% in humeral shaft fractures. In a systematic review of 21 papers with 4517 humeral shaft fractures, Shao et al. [20] reported an overall incidence of radial nerve palsy after fracture of the humeral shaft of 11.8% (532 palsies).

While treatment for radial nerve palsy is controversial within the orthopedic community, particularly regarding the necessity for early radial nerve exploration, previous studies have revealed that radial nerve palsy has a high recovery rate. Ekholm et al. [21] reported that radial nerve palsy in operative and nonoperative cohorts completely resolved in 81.8% of cases: 88.9% in the nonoperative cohort and 73.3% in the operative cohort. Shao et al. reported a recovery rate of rate radial nerve palsy of 88.1% with spontaneous recovery occurring in 70.7% of patients who were treated conservatively. The mean time to recovery for patients who spontaneously recovered was 6.1 months (range 3.4–12 months) [20].

To the best of the authors’ knowledge, few studies have globally assessed radial nerve palsy in operatively and nonoperatively treated humeral shaft fracture and delineated the recovery time in each treatment group. Furthermore, to the best of the authors’ knowledge, no study has compared the recovery time for patients with RNPc versus those with RNPi. The purpose of this study is thus twofold: (1) to comprehensively examine and report the incidence and recovery time of radial nerve palsy (RNP) in operative and nonoperative humeral shaft fracture and nonunion populations, and (2) to evaluate risk factors for developing complete and incomplete radial nerve palsy.

## Materials and methods

The fracture database of a single fellowship-trained orthopedic trauma surgeon was queried between 2007 and 2016 for patients with diaphyseal humeral shaft fracture (OTA classification type 12-A, B, C) [22, 23]. Inclusion criteria were patients aged 18 years and older with nonpathological humeral shaft fracture or nonunion. A total of 175 patients were identified and met the inclusion criteria.

Medical records were retrospectively reviewed for age, gender, demographic data, fracture type, treatment modality, operative approach, radial nerve status, presence of radial nerve exploration (if the patient was treated operatively), radial nerve palsy recovery time, and length of follow-up time from initial injury. Treatment modality, either operative or nonoperative intervention, was decided by the treating surgeon. Indications for surgery in acute fracture included open fracture, vascular injury requiring repair, and unacceptable fracture positioning (displacement and angulation: > 1 cm distraction, > 3 cm shortening, > 30° varus/valgus angulation or > 30° sagittal plan angulation) at time of injury or following initial conservative treatment based on anteroposterior (AP) and transthoracic lateral radiographic examination of the humerus [24–27].

Treatment of individual patients was decided in a shared decision-making process between surgeon and patient based upon the injury, fracture, and patient-specific characteristics. If surgery was elected, the humerus was approached by either anterolateral or posterior dissection. In all cases, the radial nerve was identified, mobilized, and protected throughout the procedure. Repair of humeral fracture or nonunion was performed using standardized techniques of either compression or bridge plating utilizing large fragment plates and screws. In all cases, care was taken to ensure that the radial nerve was free of implanted hardware and under no undo tension at the end of the procedure. If nonoperative treatment was elected, patients were kept in a coaptation plaster splint for 7–10 days, followed by conversion to a plastic functional fracture brace. In these cases, patients were seen at follow-up for repeat radiographic assessment at 2, 4, and 6 weeks to confirm maintenance of alignment.

Follow-up visits included neurological examination (sensory and motor) of the injured arm as well as assessment of range of motion of shoulder (flexion, extension, abduction, and rotation), elbow (flexion, extension, supination, and pronation), and wrist (flexion and extension). Clinical neurological examination consisted of a motor examination and a sensory examination. The motor examination used the 0–5 motor strength scale and tested the distally innervated muscles of the anterior interosseous nerve (AIN), posterior interosseous nerve (PIN), and ulnar nerve. Sensory examination of the radial, median, and ulnar nerves using light touch was performed using a cotton swab or paper clip. Nerve lesions were identified as complete (RNPc), which included motor and sensory loss, and incomplete (RNPi), which included sensory loss only. Functional recovery of the nerve was considered as return of sensory and/or motor function of the radial nerve based upon carefully documented clinical examinations.

No electromyogram (EMG) testing was employed diagnostically at time of injury. It is not our surgeons’ protocol to obtain EMG for operatively treated patients with RNP because the continuity of the radial nerve is observed directly during surgery. In our nonoperatively managed patients, it was the surgeons’ protocol to order an EMG for patients 8 weeks after RNP onset if no change was seen in radial nerve function and another in 6 months if there was no return of function.

Descriptive statistics were used to compare demographic, injury, and treatment characteristics between operative and nonoperative cohorts. Independent-samples *t*-test was used to compare nerve recovery time between cohorts. *p*-Values less than 0.05 were considered significant. Data were managed in Microsoft Excel, and statistics were analyzed using IBM SPSS.

## Results

Overall, 175 patients (84 men, 91 women) with mean age of 49.3 years (range 18–92 years) with 175 humerus fractures or nonunions and mean follow-up of 12 months (range 2–76 months) make up the initial study cohort. Two patients with RNP, one from each treatment group, were lost to final follow-up and are not included in the study analysis. Three nonunion patients had prior history of radial nerve palsy that had completely resolved before treatment of the presenting condition; these patients were included in the study analysis. Seventy-seven patients were treated operatively for either acute fracture (37 patients), symptomatic nonunion (34), or periprosthetic diaphyseal humeral shaft fracture (6). Fifty-seven underwent an anterolateral surgical approach, while 20 patients underwent a posterior surgical approach. Ninety-eight patients were treated nonoperatively for acute diaphyseal humeral shaft fracture. All fractures or nonunions in both treatment arms healed. Data regarding patient population characteristics are summarized in Table 1.

**Table 1:** Characteristics of study population

		Operative	Nonoperative
Number (%)	175	77 (44%)	98 (56%)
Age (years)	Mean: 49.3	Mean: 50.3	Mean: 48.5
	Range: 18–92	Range: 18–83	Range: 18–92
Gender	Male: 84	Male: 38	Male: 46
	Female: 91	Female: 39	Female: 52
Surgical approach	Anterolateral: 57	Anterolateral: 57	
	Posterior: 20	Posterior: 20	
Prior history of RNP (resolved)	3	3	0
Follow-up (months)	Mean: 11.9	Mean: 17.9	Mean: 6.9
	Range: 2–77	Range: 6–77	Range: 2–65
Nonunion of humerus	37	37	
Acute fracture of humerus	138	40	98
RNPc (%)	19	12 (15.6%)	7 (7.1%)
RNPi (%)	6	5 (6.5%)	1 (1.0%)
Iatrogenic RNP (%)	2	2 (3%)	

Of the 77 patients who were treated operatively, 17 (22%) were preoperatively diagnosed with a radial nerve lesion: 12 patients (15.6 %) had RNPc, and 5 patients (6%) had RNPi. All 17 underwent fracture repair with radial nerve exploration, demonstrating continuity in all cases. Of the 17 radial nerve palsy patients, 9 were treated operatively for nonunion (mean length of time from initial fracture: 16.0 ± 20.4 months, range 1–60 months), 6 for acute humeral fractures, 1 for open fracture due to gunshot wound, and 1 due to periimplant fracture. All operatively treated patients had eventual radial nerve recovery: 35.3% recovered within 2 weeks, and 64.7% recovered between 3.1 and 83.6 weeks. Mean time of functional nerve recovery for patients with RNPc in this group was 16.5 ± 25.1 weeks (median 5.3 weeks). Mean time of functional nerve recovery for patients with RNPi was 14.2 ± 9.1 weeks (median 13.4 weeks). Two (2.6%) patients treated operatively developed secondary RNPc postoperatively; both fully recovered by 24 weeks after operative treatment. One patient had evidence of early recovery beginning at 12 days but fully resolved in 27 weeks, while the other patient saw full return of function at 21 weeks.

Ninety-eight patients were treated nonoperatively for humeral shaft fracture, of whom eight patients presented with a radial nerve lesion at the time of injury: seven (7.1%) of these patients had RNPc, and one patient (1%) had RNPi. All of these patients sustained acute humeral shaft fractures that were closed; four were due to high-velocity incidents and four were due to low-velocity incidents. All patients presented with radial nerve palsy prior to any treatment, including application of a splint. Mean time of functional nerve recovery for patients with RNPc in this group was 25.2 ± 10.4 weeks (median 26.4 weeks). There was only one patient with RNPi in this group, in whom functional recovery occurred at 13.0 weeks.

Although operatively explored nerves appeared to recover more quickly, there was no statistical difference in nerve recovery time between nonoperative and operative cohorts (RNPc mean: 25.2 weeks versus 16.5 weeks; *p* = 0.433; RNPi mean: 13.0 weeks versus 14.2 weeks, *p* = 0.913). Additionally, there was no difference in nerve recovery time between RNPi and RNPc (combined operative and nonoperative cohorts, *p* = 0.541). Mean time of functional nerve recovery for patients with RNPc was 19.5 ± 21.1 weeks (median 14.3 weeks). Mean time of functional nerve recovery for patients with RNPi was 14.0 ± 8.2 weeks (median 13.4 weeks). Results data are summarized in Table 2.

**Table 2:** Nerve recovery data for complete and incomplete RNP

	*N*	Mean recovery (weeks)	Std. deviation	Significance (*p*)
RNPc recovery				
OP	12	16.5	25.1	0.433
NONOP	7	25.2	10.4	
RNPi recovery				
OP	5	14.2	9.1	0.913
NONOP	1	13.0		
Overall recovery				
RNPc	19	19.5	21.1	0.541
RNPi	6	14.0	8.2	

Logistic regression analysis revealed that age, race, gender, mechanism of injury, OTA classification, acute fracture versus nonunion and periprosthetic fracture, and surgical approach were not independent risk factors for developing RNP. Results are summarized in Table 3.

**Table 3:** Factors associated with nerve recovery

	Sig.	Odds ratio	95% CI for exp(*B*)	
			Lower	Upper
Step 1				
Gender	0.105	0.000	0.000	8.694
Age	0.083	0.749	0.540	1.039
Race	0.099	2.877	0.820	10.092
Mechanism of action	0.383	1.649	0.536	5.077
OTA classification	0.496	1.179	0.734	1.892
Acute versus nonunion versus periprosthetic Fx	0.731	0.561	0.021	15.068
Surgical approach	0.163	0.076	0.002	2.834
Constant	0.108	9,677,891.074		

CI, confidence interval

## Discussion

Radial nerve palsy has an incidence that ranges from 2 to 17% [28]. In our cohort, the overall incidence of radial nerve palsy was 15.4% (27/175), which included complete and incomplete radial nerve palsy as well as secondary radial nerve palsy. There was no difference in resolution time when patients whose fractures were managed nonoperatively were compared with those managed operatively with regard to their radial nerve palsy. However, while not statistically significantly, surgical intervention did lead to faster recovery of radial nerve palsy in the RNPc cohort. Furthermore, when recovery times of RNPc patients were compared with those of RNPi patients, regardless of surgical management, RNPi recovered slightly faster than RNPc, although not significantly so. All patients (100%), regardless of type of RNP or management type, had complete recovery of nerve function. As such, the overall radial nerve recovery rate in our study was comparable to those of previous studies, which also support a high recovery rate.

Ekholm et al. reported the incidence of radial nerve palsy associated with humeral shaft fracture as 8.5% in a population of closed humeral shaft fractures, including 361 traumatic injuries, 34 pathological fractures, and 6 periimplant fractures. In that study, there were 18 patients with radial palsy who were nonoperatively managed; 89% of these patients recovered completely, whereas the remaining 11% were left with minor sequelae from the radial nerve palsy (i.e., dysesthesia) [21]. Our study included eight nonoperatively managed humeral shaft fractures with concomitant radial nerve palsy, all (100%) of which healed clinically and radiographically. In the study by Ekholm et al., of the 15 patients managed operatively, 11 patients had complete recovery (73.3%). Of the four patients left with radial nerve palsy sequelae, two had hyperesthesia of the distal forearm and hand in the radial distribution, one patient had complete palsy, and one had incomplete palsy. Two of these patients eventually needed tendon transfers [21]. Our study found a 100% recovery rate of operatively managed patients who presented with radial nerve palsy associated with humeral shaft fracture. Their study, like ours, confirmed the high overall recovery rate of radial nerve palsy associated with humeral shaft fracture as previously reported in literature. However, their study, unlike ours, did not examine the time of recovery for each treatment group.

Shao et al., in a systematic review, also confirmed a high overall recovery rate of radial nerve palsy. They reported the overall recovery rate to be 88.1% (921 of 1045) with spontaneous recovery reaching 70.7% (411 of 581) in patients treated conservatively. The mean time to recovery for conservatively treated patients was reported to be 6.1 months, similar to the 25.2 weeks (6.3 months) reported in our study. Shao et al. [20] also found no difference in the final results when comparing groups initially managed expectantly versus those explored early, suggesting that initial expectant treatment did not adversely affect the extent of nerve recovery. Similarly, our study suggests no difference in recovery time between patients with radial nerve palsy who were managed operatively and nonoperatively. All patients in both treatment groups recovered from the radial nerve palsy, although the mean recovery time for the operative group was shorter than in the nonoperative group.

A study performed by Shah and Bhatti reviewed 62 cases of radial nerve paralysis associated with humeral shaft fracture and also reported a high rate of recovery of radial nerve palsy. Fifty-three out of 62 patients (95%) achieved normal or near-normal radial nerve function following treatment. Of the 44 injuries treated conservatively, 43 (97.7%) regained complete function of the radial nerve. Of the 12 treated surgically, 10 patients (83.3%) regained complete function of the radial nerve. This study also noted that, in 87% of patients, radial nerve recovery began less than 6 months after initial injury. For our study, this was true for 19/25 (76%) of our patients presenting with primary radial nerve palsy [27]. Shah and Bhatti also found that, of 17 patients who presented with secondary radial nerve palsy, 16 recovered completely while 1 was lost to follow-up. Our study also reflected similar findings in our experience with secondary radial nerve palsy. We had two patients with secondary radial nerve palsy, both of whom recovered complete radial nerve function [27].

This study has several limitations. Although data on treatment modality and radial nerve palsy recovery were available for all patients, this is a retrospective series and is subject to selection bias, type II error, and data collection limitations. Another limitation is the lack of EMG data for each case of nerve palsy. EMG would have the ability to discern subtle lesions of the radial nerve and may have added more patients to the RNP treatment groups for analysis. However, any positive EMG without signs and symptoms would likely be clinically insignificant. Still, this series represents a single trauma surgeon’s experience over a nearly 10-year period.

In conclusion, the results of this study further confirm the reported high overall rate of clinical recovery in patients presenting with or developing radial nerve lesion with concomitant humeral shaft injury. All 27 (100%) patients presenting with radial nerve lesion recovered, regardless of treatment modality or type of radial nerve lesion. We found that radial nerve exploration is associated with speedier resolution of RNPc when directly compared with observation as seen in the nonoperative RNPc cohort, although this was not significant. We also found no statistically significant difference in the recovery time of RNPi in operative and nonoperative cohorts. Furthermore, while not statistically significant, we found that incomplete lesions had faster recovery time when compared with complete lesions, regardless of treatment management. These findings provide surgeons with valuable information they can share with patients who sustain radial nerve injury with associated humeral shaft fracture or nonunion.
